# High prevalence of *Trypanosoma* spp. and apparent trypanocidal drugs inefficacy in cattle in Al Radom National Park, Sudan

**DOI:** 10.1038/s41598-026-37097-7

**Published:** 2026-01-27

**Authors:** Khalid M. Mohammedsalih, Maowia M. Mukhtar, Abdoelnaim I. Y. Ibrahim, Ibrahim M. I. Mohammedain, Fathel-Rahman Juma, Abdelrahim A. Ali, Elwaleed M. Elamin, Ahmed Bashar, Georg von Samson-Himmelstjerna, Jürgen Krücken

**Affiliations:** 1 Institute for Parasitology and Tropical Veterinary Medicine, Freie Universität Berlin, Robert-von-Ostertag-Str. 7, 14163 Berlin, Germany; 2 Veterinary Centre for Resistance Research, Freie Universität Berlin, 14163 Berlin, Germany; 3 Central Research Laboratory of Darfur Universities, Mousseh District, Nyala, 63311 Sudan; 4 Faculty of Veterinary Science, University of Nyala, Mousseh District, Nyala, 63311 Sudan; 5 Bioscience Research Institute, Khartoum, 11463 Sudan; 6 Institute of Endemic Diseases, University of Khartoum, Qasser Street, P.O. Box 102, Khartoum, Sudan

**Keywords:** Trypanosomosis, Trypanocidal drugs, Cattle, Al Radom National Park, Sudan, Infectious diseases, Therapeutics, Parasitology, Infectious-disease diagnostics, Epidemiology

## Abstract

Trypanosomosis is a major infectious disease affecting cattle in Sudan. In South Darfur, data on cattle trypanosomosis has been limited since the Darfur civil war (2003–2007). This study assessed the prevalence and distribution of *Trypanosoma* spp. in cattle in the tsetse fly endemic Al Radom National Park, and collected questionnaire data on trypanocide use and apparent efficacy. Blood from 509 cattle across four regions was analysed. Of these, 3.1%, 19.8% and 35.8% tested positive for trypanosomes by microscopy, buffy coat technique and PCR, respectively. At the regional level, prevalence was 50.0%, 42.9%, 27.3% and 22.9% in Al Radom Livestock Market, Kafindibei, Al Radom town and Murayrayah, respectively. *Trypanosoma congolense* savannah (15.7%), *Trypanosoma vivax* (9.6%), *Trypanosoma brucei* (0.8%) and *Trypanosoma theileri* (13.4%) were identified. Prevalence was significantly correlated with region and age (*P* < 0.05). Older cattle showed significantly higher prevalence (44.7%) than 1–3 years old (29.7%) and < 1 year old cattle (22.7%). Most cattle (86.8%) had received trypanocides within 30 days before sample collection, mainly diminazene aceturate, either alone or combined with isometamidium chloride and/or quinapyramine. Despite treatment, 30.7% were *Trypanosoma*-positive by PCR. In conclusion, trypanosomes are prevalent in Al Radom National Park, even in treated cattle, indicating apparent drug inefficacy, which requires further research and control measures.

**Supplementary Information:**

The online version contains supplementary material available at 10.1038/s41598-026-37097-7.

## Introduction

Trypanosomosis is a disease caused by unicellular *Trypanosoma* parasites found in the blood and other tissues of vertebrates^1^. In cattle, in order of importance, *Trypanosoma congolense*, *Trypanosoma vivax*, *Trypanosoma brucei* and *Trypanosoma evansi* are identified as more virulent. However, other species such as *Trypanosoma theileri* and *Trypanosoma simiae*, which primarily infects pigs but can also occur in cattle, are considered to have a lower pathogenic impact^2^. Genotypic characterisation of *Trypanosoma* species has identified various subspecies. *Trypanosoma congolense* has been genotyped into four subspecies, including *T. congolense* “forest type” (west African riverine), *T. congolense* “savannah type”, *T. congolense* “Kilifi type” and *T. congolense* “Tsavo type”^3–5^. Pathogenicity can vary between the subspecies, and experimental infection studies have shown differences in virulence between the *T. congolense* forest, savannah and Kilifi subspecies in mice and cattle, with *T. congolense* savannah being the most pathogenic^3,4^.

*Trypanosoma* spp. can be transmitted biologically by tsetse flies (genus *Glossina*), including species such as *T. congolense*, *T. vivax*, *T. brucei* and *T. simiae*. These species are known as Salivarian trypanosomes, transmitted via the saliva of tsetse flies (*Glossina* spp.), and are responsible for causing African Animal Trypanosomosis^6^. However, *T. evansi* is only transmitted mechanically through the mouthparts of other blood-feeding dipterans such as tabanids, *Stomoxys* spp., keds, mosquitoes and sand flies^7^. *Trypanosoma theileri* uses biological transmission with tabanids as intermediate hosts. In many regions, biological and mechanical transmission of *Trypanosoma* species co-exist in the same hosts and sometimes even involve the same vectors^8^.

Trypanosomosis in cattle is characterised by anorexia, weakness, fever, pale mucous membranes, weight loss, neurological symptoms and potentially death^2^. The resulting economic losses are estimated at $4.5 billion annually^9^. Subclinically infected livestock (e.g. cattle, sheep and goats) and wildlife serve as reservoirs^2^. The subclinical nature of symptoms and low parasite levels often complicate diagnosis via clinical observation or routine blood smears. Therefore, PCR has been proposed as a sensitive and accurate diagnostic tool, especially for subclinical cases^10^. However, the pan-species nested PCR targeting the ribosomal internal transcribed spacer (ITS) region described by Cox, et al.^11^ has been widely used due to its ability to distinguish all clinically important *Trypanosoma* species and some subspecies in a nested PCR reaction, reducing both time and cost. This technique has been used for detecting *Trypanosoma* species in livestock and wildlife in sub-Saharan Africa, including Sudan^12,13^. For robust species and subspecies identification, the use of species-specific PCRs is also crucial to produce more detailed epidemiology data. Species-specific PCRs are well established for detecting *T. congolense* forest type, *T. congolense* savannah, *T. vivax*, *T. brucei* sensu lato, *T. brucei gambiense*, *T. evansi*, *T. simiae* and *T. theileri*^5,14–19^.

Control measures for animal trypanosomosis include vector control, chemotherapy and breeding resistant livestock, yet the disease continues to impact livestock health and rural economies in the region^1^. However, for over a century, the control has depended largely on chemotherapy, particularly relying on the use of four trypanocide drugs: diminazene aceturate (Berenil), melarsamine hydrochloride (cymelarsan), quinapyramine compounds (Antrycide) and isometamidium chloride (Samorin). Their application, especially in recent years, has been associated with the development of resistance, which is reducing their effectiveness and raising concerns about future treatment options^20^.

Diminazene aceturate has been widely used to treat animal trypanosomosis for over 60 years. It is known for its rapid excretion and popular due to its high therapeutic index, low resistance incidence and affordability. However, its protective effect is short-lived, typically lasting up to three weeks^21^. Melarsamine hydrochloride is particularly effective against *T. evansi* in camels and other livestock. It is useful against Berenil-resistant strains, including *T. evansi* and *T. brucei*. This drug also enhances treatment outcomes when combined with other trypanocides^22^. Quinapyramine, a bis-quaternary ammonium compound introduced in the 1950s, is available as quinapyramine sulphate (Antrycide Sulphate) for therapeutic and quinapyramine chloride (Antrycide Prosalt) for prophylactic use. These compounds are effective against *T. congolense*, *T. vivax* and *T. brucei* in cattle, sheep and goats. Combined, quinapyramine chloride and sulphate offer chemoprophylactic protection for three to four months^21,23^. Isometamidium chloride, a derivative of homidium and part of the diminazene aceturate molecule, is also used for both preventive and therapeutic purposes^24^. After intramuscular administration in cattle, its prophylactic effect usually lasts two to three months, but can be extended up to six months, depending on factors like formulation, dosage, *Trypanosoma* species and the animal’s health^23^.

In Sudan, trypanosomosis has been identified in domestic ruminants using parasitological methods (such as blood smear staining and the buffy coat technique), serology and molecular approaches^25–28^. The pathogenic trypanosomes, *T. congolense*, *T. vivax* and *T. brucei*, have been found in cattle, sheep and goats, particularly near South Sudan, including in Blue Nile, East Darfur and South Darfur states. *Trypanosoma evansi* and *T. vivax* have been identified in dromedary camels^26^. The widespread presence of trypanosome vectors^29^ continues to challenge livestock farming in Sudan. South Darfur State, with an estimated 2.4 million cattle, is vital for rural livelihoods and the national economy^30^. However, data on animal trypanosomosis are limited, especially after the Darfur civil war (2003–2007). This study aimed to fill this gap by providing epidemiological data on *Trypanosoma* spp. infections in cattle at Al Radom National Park using parasitological and molecular techniques. Additionally, a questionnaire survey was conducted on the use of trypanocides in cattle in the park area.

## Materials and methods

### Study area

This study was conducted in Al Radom National Park (9.85°N, 24.83°E) in southwestern South Darfur State, Sudan, near the borders with South Sudan and the Central African Republic, where tsetse flies are endemic^29^ (Supplementary Fig. 1). The park covers about 1,250,970 hectares of high-rainfall wooded savannah. Rainfall occurs from May to November, peaking between July and September, with annual totals of 900 to 1,700 mm. Mean annual humidity ranges from 57% to 65% and temperatures vary from 16 °C to 31 °C. During the dry season (December to April), temperatures are usually below 36 °C, though some precipitation can occur^31^. Nomadic communities migrate to the area with their livestock during this period and may occasionally move to South Sudan towards the end of the dry season (March to May), depending on rainfall. As the rainy season begins, they migrate northward. The study focused on three different regions: Al Radom town (9.55°N, 24.59°E), Kafindibei (9.85°N, 24.49°E) and Murayrayah (9.87°N, 24.95°E).

### Study animals

The study focused on indigenous *Baggara* cattle, an East African shorthorn zebu breed (*Bos taurus indicus*).

### Study design, sample size and sampling strategies

A cross-sectional survey was conducted from January to February 2020 to evaluate the prevalence of trypanosomosis and to assess the use and apparent efficacy of trypanocidal drugs in cattle. Three regions were selected: Al Radom town, Kafindibei and Murayrayah. The strategy for including the region was based on the watercourse availability, forest density and livestock density. First, we selected a central point (Al Radom town) (Supplementary Fig. 1). Thereafter, to avoid the factor of shared pasture shortly before sample collection, and based on nomads’ movement directions, a minimum distance of 25 km between the regions was intended. Therefore, in addition to the central point, two regions in opposite directions from Al Radom were selected: one characterised by low watercourse availability and low forest density (Murayrayah, 36 km northeast of Al Radom), and the other by high watercourse availability and high forest density (Kafindibei, 35 km southwest of Al Radom near the South Sudan border) (Supplementary Fig. 1). Kafindibei’s climate supports a higher abundance of tsetse flies compared to the other two regions^29^. In Al Radom, samples were collected from local farms and the Al Radom Livestock Market. The Livestock Market samples can provide information on *Trypanosoma* spp. in cattle from surrounding villages; however, bias in the results can be expected due to the selective nature of nomadic cattle marketing practices (e.g. animal age and health status)^32^.

The required sample size was calculated using Epitools epidemiological calculators^33^, with a confidence level of 95%, a desired precision of 0.04 and an estimated true proportion of 0.3.

The Veterinary Research Laboratory at Al Radom assisted with a nomadic guide familiar with the region and local nomadic communities. In each of the four regions, the guide provided directions to the herds, either in Al Radom town and at the Livestock Market, or at the livestock collection points at Murayrayah and Kafindibei, which are locally known as “*fariq*”. These points typically comprise groups of animals from various species (primarily cattle, sheep and goats) owned by a single individual or nomadic family. In each *fariq* (herd), cattle were selected using convenience sampling. To better manage unrestrained cattle, samples were collected in the evening (6:00 to 10:00 PM, GMT + 2), with 50% to 100% of the cattle in each *fariq* (herd) sampled. At the Al Radom Livestock Market, samples were collected around midday from five different herd groups, each representing one of the surrounding villages/*fariqs* near Al Radom.

### Sample collection and processing

Blood samples were collected from the jugular vein of female and male cattle. The animals, representing various age groups (< 1 year, 1–3 years and > 3 years, as classified by dentition according to Saini, et al.^34^, were derived from different herds (*fariqs*)). The distribution of samples across the study regions was as follows: 33 cattle from Al Radom town (3 herds), 38 from the Al Radom Livestock Market (5 villages*/fariqs*), 170 from Murayrayah (9 *fariqs*) and 268 from Kafindibei (10 *fariqs*). From each animal, 10 ml EDTA/blood was collected for direct parasitological examination and for storage at -20 °C for molecular analysis.

### Questionnaire survey

A pretested semi-structured questionnaire was administered to collect data on knowledge of trypanosomosis, tsetse flies, risk factors for *Trypanosoma* spp. infection and the use of trypanocidal drugs (diminazene aceturate, isometamidium chloride, melarsamine hydrochloride and quinapyramine sulphate and chloride). The questionnaire also explored treatment strategies, including the use of these four drugs either alone or in combination. Interviews were conducted with nomads, each owning a distinct herd/*fariq*, distributed as follows: three from Al Radom town, five from the Al Radom Livestock Market, nine from Murayrayah and 10 from Kafindibei. The chemoprophylactic effectiveness of the four trypanocides is known to range from approximately 3 weeks for diminazene aceturate to 2–4 months for isometamidium chloride and quinapyramine sulphate and chloride^21–23^. The life cycle of *Trypanosoma* spp. begins when *Glossina* spp. inject metacyclic trypomastigotes into the animal, taking one to four weeks to complete, depending on factors such as the animal’s health^35^. In South Darfur State, nomads usually treat their animals, such as with anthelmintics^36^, every two weeks or once per month. With these three factors in mind, we used a 30-day period prior to sample collection to obtain information on the last trypanocides administered within this timeframe. This allowed us to collect data on the frequency of trypanocidal drug use and the apparent chemoprophylactic effectiveness, whether the drugs were administered alone or in combination. For diminazene aceturate and melarsamine hydrochloride, when administered alone, the 30-day period provided some information on apparent effectiveness but it can also provide data on the reinfection.

### Parasitological examination for *Trypanosoma* spp.

#### Blood smears

Thin blood smears were prepared directly in the field and fixed with methanol for 5 min. In the laboratory, the smears were stained with 10% Giemsa solution for 45 min, then washed with distilled water and air-dried. The slides were examined at 1000× total magnification (oil immersion). Each sample was analysed by one of two trained laboratory technicians who meticulously examined the entire smeared area on each slide for the presence of *Trypanosoma* spp. following standardised protocols^37^.

### Buffy coat technique (BCT)

The packed cell volume (PCV) of blood samples was assessed using centrifugation in glass capillaries in replicates, and then the PCV mean of each sample was calculated. Cattle with a PCV < 24% were classified as anaemic, all others were deemed non-anaemic^38^. Following PCV measurement, the two capillary tubes from each animal’s blood sample were examined under a light microscope at 400× magnification to identify motile trypanosomes^39^. Each animal was considered positive for *Trypanosoma* spp. if motile trypanosomes were detected in either of the two capillary tubes.

### Molecular detection of ***Trypanosoma*** spp.

#### DNA extraction

Genomic DNA was extracted using the G-spin™ Total DNA Extraction Kit, following the protocol provided by the manufacturer (iNtRON Biotechnology, Seongnam, South Korea). The DNA was eluted in 50 µl of elution buffer. The concentration and purity of the DNA were assessed using a NanoDrop™ 2000/2000c Spectrophotometer (Thermo Fisher Scientific, Waltham, MA, USA). The DNA samples were then stored at -20 °C until further analysis.

### Polymerase chain reactions

Genomic DNA samples were screened for *Trypanosoma* spp. using nested ITS primers as described by Cox, et al.^11^, using facilities available at the Bioscience Research Institute, Khartoum, Sudan. These primers amplify regions of a partial 18 S, ITS1, 5.8 S, ITS2 and a partial 28 S gene, with respective band sizes detailed in Supplementary Table 1. A nested PCR approach was used, first with outer primers ITS1 and ITS2, followed by inner primers ITS3 and ITS4 (Supplementary Table 1). PCRs were performed in 20 µl volumes containing 2 µl genomic DNA (~ 40 ng), 10 pmol primers in 20 µl of 1× PCR Master Mix (Maxime™ PCR PreMix Kit (i-MAX II), iNtRON Biotechnology) on a Bio-Rad T100 thermal cycler. The thermal profile was: initial denaturation at 95 °C for 7 min, 35 cycles of denaturation at 94 °C for 60 s, annealing at 55 °C for 60 s and extension at 72 °C for 120 s. The second PCR used 1 µl of the first-round product with inner primers ITS3 and ITS4 (Supplementary Table 1), applying the same Master Mix and cycling conditions. Second-round PCR products were analysed on 1.5% agarose gels stained with RedSafe™ Nucleic Acid Staining Solution (iNtRON Biotechnology) and visualised under ultraviolet transillumination (NuGenius, Syngene, Cambridge, UK).

Samples showing the characteristic band size for each or more of the six *Trypanosoma* species/subspecies^11^ were subjected to another round of PCR with species-specific primers according to the Supplementary Table 1. The species-specific PCRs were performed at the Institute for Parasitology and Tropical Veterinary Medicine, FU Berlin, Germany. In situations where more than one band was detected in a sample using the nested ITS primers, the corresponding *Trypanosoma* species at the nested ITS primers level were first identified based on the band sizes, as detailed in Supplementary Table 1. Then, species-specific primers were used for further confirmation of the respective *Trypanosoma* species. Since the band sizes for *T. simiae*, *T. simiae* Tsavo and *T. theileri* fall between 847 and 998 bp (nested ITS primers), any samples with bands in this range were PCR tested with both *T. simiae* and *T. theileri* species-specific primers (Supplementary Table 1). Due to extensive genetic similarity between *T. brucei* and *T. evansi*^40^, samples showing band size corresponding to *T. brucei* when using nested ITS primers (1215 bp, Supplementary Table 1) were further examined by PCR using species-specific primers for *T. brucei* sensu lato and *T. brucei gambiense*, and also a species-specific primer for *T. evansi* (Supplementary Table 1). The PCR reactions were performed in 20 µl volumes containing 2 µl genomic DNA (~ 40 ng), 10 pmol of each primer and 10 µl of 2× GoTaq^®^ qPCR Master Mix (Promega Corporation, Madison, USA) on a Genetouch thermocycler (Bioer Technology, Hangzhou, China) with conditions: initial denaturation at 95 °C for 2 min, 35 cycles of denaturation at 95 °C for 15 s, annealing at the primer-specific annealing temperature (Supplementary Table 1) for 30 s, and extension at 72 °C for 30 s. Positive and negative controls were included in each set of reactions. The laboratory had positive control samples for *T. vivax*. For other *Trypanosoma* species (*T. congolense* forest type (GenBank: S50876), *T. congolense* savannah (GenBank: M30391), *T. brucei* sensu lato (GenBank: K00392), *T. brucei gambiense* (GenBank: FN555990), *T. evansi* (derived from GenBank: M81594 but slightly changed to reduce complexity and allow synthesis), *T. simiae* (GenBank: S50874) and *T. theileri* (GenBank: LC438510)), synthetic DNA fragments were used as positive controls (5 copies per reaction), paired with species-specific primers (Supplementary Table 1) to ensure sensitivity of PCR testing. These synthetic DNA fragments (G blocks) were produced by Integrated DNA Technologies (Leuven, Belgium). PCR products, four samples representing each *Trypanosoma* species, were purified using the DNA Clean & Concentrator™ kit (Zymo Research, Freiburg, Germany) and Sanger sequenced by LGC Genomics (Berlin, Germany). Results were analysed using BLASTn^41^.

### Statistical analysis

The data from the questionnaire and the survey on *Trypanosoma* spp. infections of cattle from four selected regions in Al Radom National Park were analysed using R software version 4.4.1 and RStudio version 2024.04.2. Descriptive statistics were performed using the summarytools package version 1.0.1 to analyse the questionnaire, PCV and prevalence data. The 95% confidence intervals (CIs) for proportions in the questionnaire data and *Trypanosoma* spp. prevalence were calculated using Wilson score intervals via the binom.wilson() function from the epitools package version 0.5–10.1. Frequencies of positive samples and frequency of co-infections (compared to theoretical values calculated from the individual species frequencies) were compared using the mid-p exact test (Table 2by2() from epitools). To assess the agreement between the three laboratory techniques used for detecting *Trypanosoma* spp. infections (microscopic examination of Giemsa-stained blood smear, buffy coat technique and molecular technique), Cohen’s κ coefficients were calculated with the CohenKappa() function in the DescTools package version 0.99.55, with 95% CI. Interpretation of Cohen’s κ values followed McHugh^42^: perfect agreement (1), near perfect (0.81–0.99), substantial (0.61–0.80), moderate (0.41–0.60), fair (0.21–0.40), slight (0.0–0.20) and no agreement (< 0.0). To investigate factors affecting the prevalence of *Trypanosoma* spp. in cattle in Al Radom National Park, a generalized linear mixed model (GLMM) analysis was applied using the glmmTMB package (version 1.1.10)^43^. Both bivariable and multivariable models were used, with the exploratory variables being: study region (Murayrayah vs. Al Radom town, Al Radom Livestock Market and Kafindibei), sex (male vs. female), age (young (less than one year) vs. 1–3 years and more than three years old^34^, PCV (anaemic vs. non-anaemic^38^, and trypanocidal drug treatments (treated vs. untreated). The unique herd/*fariq* was included as a random effect. Moreover, factors associated with trypanocidal drug treatments were calculated using GLMM, both bivariable and multivariable models, with the variables being sex (male vs. female), age (young vs. 1–3 years and more than three years old), and PCV (anaemic vs. non-anaemic). Herd/*fariq* was included as a random effect. Furthermore, factors associated with PCV were also calculated using GLMM, both bivariable and multivariable models, with the variables being study region (Murayrayah vs. Al Radom town, Al Radom Livestock Market and Kafindibei), sex (male vs. female), age (young vs. 1–3 years and more than three years old), trypanocidal drug treatments (treated vs. untreated), and *Trypanosoma* spp. infection (non-infected vs. infected). Again, herd/*fariq* was included as a random effect variable. Stepwise elimination of variables was conducted using the drop1 function to minimise the Akaike information criterion (AIC). The estimates were then exponentiated and the tidy() function from the broom.mixed package (version 0.2.9.6) was applied to model coefficients to calculate the odds ratios with 95% CI. The goodness-of-fit was assessed using the conditional R² from the performance package (version 0.12.3), which estimates the proportion of variance in the response variable explained by both fixed and random factors^44^. Significance was considered at *P* < 0.05.

### Ethics statement

The samples were collected by qualified veterinarians using standard sample collection techniques, ensuring no injury or stress to the cattle. Nomads were always informed about the study’s objectives and the nature of the analyses. All animal procedures were conducted in accordance with the Sudanese Animal Welfare Law, approved in 2015, and the study received ethical approval from the Research and Ethics Committee of the Faculty of Veterinary Science, University of Nyala, Sudan (Reference No. UN/FVS/1/20/1). Since many of the nomads were illiterate, informed verbal consent was obtained for their participation in the study. This process was approved by the Ethics Committee. The related methods are reported in compliance with the ARRIVE (Animal Research Reporting of In Vivo Experiments) guidelines.

## Results

### Demographic profile of the study population and key insights from questionnaire findings

The study interviewed 27 nomads, each representing a different herd or *fariq*, with 5 to 53 cattle (median: 13), across four regions in Al Radom National Park: three from Al Radom town, five from the Al Radom Livestock Market, nine from Murayrayah and 10 from Kafindibei. The nomadic system was the most common husbandry practice (24/27 respondents (88.9%)) while sedentary farming was rarely used (3/27 (11.1%)) (Supplementary Table 2). The majority of respondents (24/27 (88.9%)) reported encounters between their livestock and wildlife, primarily in grazing areas. All nomads were familiar with tsetse flies (27/27 (100%)), and 51.9% had seen the fly within the six months before the interview. All 27 interviewed nomads were familiar with trypanosomosis and aware that tsetse flies are the primary vectors. The results indicated that 100% of respondents practised self-diagnosis for trypanosomosis and all of them mentioned more than two symptoms from the list provided, as explained in the Supplementary Table 2. Moreover, 100% of the respondents were aware that the use of trypanocidal drugs and the control of tsetse flies, through methods such as changing the pasture, cleaning shelters and burning dung, using smoke from wood or burning dung, and/or applying pour-on insecticides, are the main treatment and control methods for trypanosomosis (Supplementary Table 2).

The study involved 509 cattle, 6.5% in Al Radom town, 7.5% in the Al Radom Livestock Market, 33.4% in Murayrayah and 52.7% in Kafindibei. Of these animals, 55.4% were over three years old and females represented 73.1% (Table 1).

**Table 1:** Prevalence of *Trypanosoma* spp. infection, possible associated demographic risk factors, and the use of trypanocidal drugs, based on a questionnaire survey from naturally infected cattle in Al Radom National Park, Sudan.

Factor	All animals	Sex		Age group			Study region			
		Male	Female	< 1 year	1–3 years	> 3 years	Al Radom town	Al Radom Livestock Market	Kafindibei	Murayrayah
No. of the tested cattle	509									
No. (%) by category^a^	–	137 (26.9)	372 (73.1)	163 (32.0)	64 (12.6)	282 (55.4)	33 (6.5)	38 (7.5)	268 (52.7)	170 (33.4)
No. (%; 95% CI) of the infected cattle^b^	182 (35.8; 31.7–40.0)	50 (36.5; 28.9–44.8)	132 (35.5; 30.8–40.5)	37 (22.7; 16.9–29.7)	19 (29.7; 19.9–41.8)	126 (44.7; 39.0–50.5)	9 (27.3; 15.1–44.2)	19 (50.0; 38.9–65.2)	115 (42.9; 37.1–48.9)	39 (22.9; 17.3–29.8)
PCR	182 (35.8; 31.7–40.0)	50 (36.5; 28.9–44.8)	132 (35.5; 30.8–40.5)	37 (22.7; 16.9–29.7)	19 (29.7; 19.9–41.8)	126 (44.7; 39.0–50.5)	9 (27.3; 15.1–44.2)	19 (50.0; 38.9–65.2)	115 (42.9; 37.1–48.9)	39 (22.9; 17.3–29.8)
Buffy coat technique	101 (19.8; 16.6–23.5)	26 (19.0; 13.3–26.4)	75 (20.2; 16.4–24.5)	19 (11.7; 5.6–17.5)	6 (9.4; 4.4–19.0)	76 (27.0; 22.1–32.4)	3 (9.1; 3.1–23.6)	4 (10.5; 4.2–24.1)	90 (33.6; 28.2–39.4)	4 (2.4; 0.9–5.9)
Microscopic examination	16 (3.1; 1.9–5.1)	6 (4.4; 2.0–9.2)	10 (2.7; 1.5–4.9)	1 (0.6; 0.1–3.4)	0 (0.0; 0.0–5.7)	15 (5.3; 3.3–8.6)	1 (3.1; 0.5–15.3)	2 (5.3; 1.5–17.3)	13 (4.9; 2.9–8.1)	0 (0.0; 0.0–2.2)
No. (%; 95% CI) of cattle received trypanocides^c^	442 (86.8; 83.6–89.5)	113 (82.5; 75.3–87.9)	329 (88.4; 84.8–91.3)	142 (87.1; 81.1–91.4)	47 (73.4; 61.5–82.7)	253 (89.7; 85.6–92.7)	7 (21.2; 10.7–37.8)	1 (2.6; 0.5–13.5)	268 (100; 98.6–100)	166 (97.7; 94.1–99.1)
No. (%; 95% CI) of cattle received trypanocides and were infected	156 (30.7; 26.8–34.8)	39 (28.5; 21.6–36.5)	117 (31.5; 26.9–36.3)	31 (19.0; 13.7–25.7)	11 (17.2; 9.9–28.2)	114 (40.4; 34.9–46.3)	2 (6.1; 1.7–19.6)	1 (2.6; 0.5–13.5)	115 (42.9; 37.1–48.9)	38 (22.4; 16.7–29.2)
PCV (%) of the tested cattle: Mean ± SD	28.8 ± 5.9	27.3 ± 5.9	29.3 ± 5.8	27.6 ± 5.8	29.8 ± 6.3	29.2 ± 5.7	33.6 ± 5.8	27.6 ± 4.6	28.9 ± 5.1	28.0 ± 6.7
PCV (%) of the infected cattle: Mean ± SD	29.3 ± 5.9	27.7 ± 5.8	30.0 ± 5.8	28.5 ± 6.3	32.1 ± 7.0	29.2 ± 5.5	35.4 ± 7.0	28.3 ± 5.1	28.9 ± 5.1	29.8 ± 7.2

*95% CI* 95% confidence interval (lower and upper limit), *SD* standard deviation, *PCV* packed cell volume.

^a^Percentages (%) for this raw were calculated from the total number of animals (*n* = 509).

^b^Cattle were considered positive for *Trypanosoma* spp. if they tested positive by any of the three diagnostic techniques: microscopic examination, buffy coat and PCR.

^c^Cattle treatment with trypanocide drugs within the 30 days preceding sample collection was determined based on a questionnaire survey.

### Prevalence of *Trypanosoma* spp. infections

The overall infection rate was 35.8% (95% CI: 31.7–40.0%) (Table 1). Cattle were considered positive for *Trypanosoma* spp. if they tested positive by any of the three diagnostic techniques: microscopic examination, buffy coat and molecular techniques. The infection rate among females (35.5%, 132/372) was slightly lower than in the few included males (36.5%, 50/137). Among the animal ages, cattle over three years old had the highest prevalence (44.7%), while those aged 1–3 years and younger than one year had lower rates of 29.7% and 22.7%, respectively. Prevalence of *Trypanosoma* spp. in cattle in Al Radom Livestock Market was 50.0% and higher compared to other regions, where prevalence ranged from 22.9% to 42.9% (Table 1). Bivariable and multivariable generalized linear mixed logistic regression models were calculated with herd/*fariq* included as a random effect (Table 2). The study region and age group of the animals had significant effects (*P* < 0.05) in both types of models on the odds of cattle being positive for *Trypanosoma* spp. In contrast, the variables sex, PCV and trypanocidal drug treatments were not significant (Table 2). Cattle from Al Radom Livestock Market (odds ratio: 3.500 and 3.714 for bivariable and multivariable models, respectively) and Kafindibei (odds ratio: 2.841 and 2.659 for bivariable and multivariable models, respectively) had significantly higher infection rates with *Trypanosoma* spp. compared to those from Murayrayah (*P* < 0.05). Infections were significantly more frequent in animals older than three years compared to those aged less than one year (odds ratio: 2.697 and 3.266 for bivariable and multivariable models, respectively, *P* < 0.001). However, the middle-aged group (1–3 years) showed non-significant P-values of 0.448 and 0.329 for bivariable and multivariable models, respectively (Table 2).

**Table 2:** Final logistic regression mixed model identifying variables influencing *Trypanosoma* spp. infections in the blood of naturally infected cattle from Al Radom National Park, Sudan, using herd/*fariq* as a random effect variable.

	Bivariable^a^			Multivariable^b^		
	Odds ratio	95% CI	*P* value	Odds ratio	95% CI	*P* value
Study region; Murayrayah vs.						
Al Radom town	1.326	0.473–3.718	0.591	1.399	0.511–3.832	0.513
Al Radom Livestock Market	3.500	1.152–10.630	0.027	3.714	1.309–10.539	0.014
Kafindibei	2.841	1.575–5.127	0.001	2.659	1.514–4.668	0.001
Sex; male vs.						
Female	0.958	0.618–1.487	0.850	0.614	0.373–1.011	0.055
Age; young (< 1 year) vs.						
1–3 years	1.307	0.655–2.611	0.448	1.421	0.702–2.880	0.329
> 3 years	2.697	1.709–4.254	< 0.001	3.266	1.997–5.339	< 0.001
PCV; non-anaemic (≥ 24%) vs.						
Anaemic (< 24%)	1.117	0.630–1.981	0.706	1.250	0.691–2.260	0.461
Trypanocide use; treated vs.						
None	1.146	0.490–2.68	0.754			

^a^ Conditional Nakagawa R^2^ (range): 0.106–0.138; Marginal Nakagawa R^2^ (range): 0.000–0.070.

^b^Conditional Nakagawa R^2^: 0.160; Marginal Nakagawa R^2^: 0.132.

PCR, using pan-*Trypanosoma* primers identified a significantly (*P* < 0.05, mid-p exact test) higher proportion of positive samples (35.8%; 95% CI: 31.7–40.0%) compared to the buffy coat technique (19.8%; 95% CI: 16.6–23.5%) and the microscopic examination (3.1%; 95% CI: 1.9–5.1%) (Table 1). Among the three techniques, PCR and the buffy coat technique showed substantial agreement (Cohen’s κ value: 0.62; 95% CI: 0.55–0.67). In contrast, the agreement between the microscopic examination and the other two techniques was only fair to slight (Table 3).

**Table 3:** Agreement among the three techniques used in the detection of *Trypanosoma* spp. infections in the blood of naturally infected cattle from Al Radom National Park, Sudan.

	Microscopic examination		Buffy coat		PCR		Cohen’s κ (95% CI)	
	Positive	Negative	Positive	Negative	Positive	Negative	Microscopic examination	Buffy coat
Microscopic examination								
Positive	16	n.a.	16	0	16	0		
Negative	n.a.	493	85	408	166	327		
Buffy coat								
Positive	16	85	101	n.a.	101	0	0.23 (0.14–0.33)	
Negative	0	493	n.a.	408	81	327		
PCR								
Positive	16	166	101	81	182	n.a.	0.11 (0.06–0.16)	0.62 (0.55–0.67)
Negative	0	327	0	327	n.a.	327		

*95% CI* 95% confidence interval (upper and lower limit), *n.a.* not available.

### Identification of *Trypanosoma* species and their prevalence based on PCR

Although the amplicon size differs between different *Trypanosoma* spp. in the initial ITS pan-*Trypanosoma* PCR (Supplementary Table 1), species assignment purely based on amplicon size was considered to be not reliable enough since the large number of potentially occurring *Trypanosoma* spp. made it difficult to assign a fragment unequivocally to a species. Therefore, all samples positive in the pan-*Trypanosoma* PCR were further analysed using species-specific primers. Additionally, selected PCR products (*n* = 4 for each same amplicon size) were sequenced. In total, four *Trypanosoma* species were identified in cattle at Al Radom National Park: *T. congolense*, *T. vivax*, *T. brucei* and *T. theileri*. The obtained sequences were 100% identical to *T. vivax* (GenBank HE573027), 98.0% identical to *T. congolense* savannah (GenBank M30391) and 100% identical to *T. theileri* (GenBank AB930155). However, for *T. brucei*, a nested pan-*Trypanosoma* PCR detected four bands at the expected size (1215 bp), but none of the three species-specific *T. brucei* primers, as well as the species-specific *T. evansi* primer (Supplementary Table 1), produced results. Thus, the nested pan-*Trypanosoma* PCR was the only method to detect *T. brucei*. The samples were considered positive in the following, but the results should be considered with care. Additional investigations were unfortunately not possible since the sample material was too limited.

The total prevalence of *Trypanosoma* spp. infections was reported at 35.8% (95% CI: 31.7–40.0%), with *T. congolense* savannah being the most frequent species (15.7%; 95% CI: 12.8–19.1%), followed by *T. theileri* at 13.4% (95% CI: 10.7–16.6%). *Trypanosoma vivax* was detected in 9.6% of the cattle, while *T. brucei* had the lowest prevalence, being detected in only four animals (Table 4). Coinfections with two *Trypanosoma* species, but not more, did occur in 3.7% (95% CI: 2.4–5.8%) of the animals, with coinfections of *T. vivax* and *T. theileri* being the most frequent at 2.8% (95% CI: 1.7–4.6%) (Table 4). A similar prevalence of *T. congolense* savannah was detected in males (15.3%) and in females (15.9%). However, female cattle showed a higher prevalence of 13.4% for *T. theileri* compared to 3.5% in males. Cattle over three years old exhibited 24.8% (95% CI: 20.1–30.2%) *T. congolense* savannah as the highest prevalence among different *Trypanosoma* species, compared to 5.5% and 1.6% for younger animals and 1–3 years old, respectively. Additionally, female and older cattle showed more positive cases of coinfections (Table 4). Among the four study regions, the prevalence of the four *Trypanosoma* species was higher in Al Radom Livestock Market compared to the other three regions, while a higher coinfection rate was found in Murayrayah (4.7%) (Table 5). For coinfections of *T. vivax* and *T. theileri*, the highest prevalence was reported in Murayrayah at 4.1% (Table 5). None of these coinfections were significantly more or less frequently found than expected from the individual prevalences of the species (mid-p exact test and correction for multiple testing using the Holm method, *P* > 0.05).

**Table 4:** Prevalence, number and percentage of *Trypanosoma* spp. in the blood of naturally infected cattle of different sexes and age groups in Al Radom National Park, Sudan, detected using PCR.

Prevalence	All animals		Sex				Age group					
			Male		Female		< 1 year		1–3 years		> 3 years	
	*n*	% (95% CI)	*n*	% (95% CI)	*n*	% (95% CI)	*n*	% (95% CI)	*n*	% (95% CI)	*n*	% (95% CI)
Tested cattle	509	–										
No. by category			137	–	372	–	163	–	64	–	282	–
Total infection^a^	182	35.8 (31.7–40.0)	50	36.5 (28.9–44.8)	132	35.5 (30.8–40.5)	37	22.7 (16.9–29.7)	19	29.7 (19.9–41.8)	126	44.7 (39.0–50.5)
*T. brucei* ^b, c^	4	0.8 (0.3–2.0)	1	0.7 (0.1–4.0)	3	0.8 (0.3–2.3)	1	0.6 (0.1–3.4)	0	0.0 (0.0–5.7)	3	1.1 (0.4–3.1)
*T. congolense* savannah^b, c^	80	15.7 (12.8–19.1)	21	15.3 (10.3–22.3)	59	15.9 (12.5–19.9)	9	5.5 (2.9–10.2)	1	1.6 (0.3–8.3)	70	24.8 (20.1–30.2)
*T. theileri* ^b, c^	68	13.4 (10.7–16.6)	18	3.5 (13.1–19.8)	50	13.4 (10.4–17.3)	16	9.8 (6.1–15.4)	12	18.6 (11.1–30.0)	40	14.2 (10.6–18.7)
*T. vivax* ^b, c^	49	9.6 (7.4–12.5)	14	10.2 (6.2–16.4)	35	9.4 (6.8–12.8)	14	8.6 (5.2–13.9)	8	12.5 (6.5–22.8)	27	9.6 (6.7–13.6)
Coinfection species	19	3.7 (2.4–5.8)	4	2.9 (1.1–7.3)	15	4.0 (2.5–6.6)	3	1.8 (0.6–5.3)	2	3.1 (0.9–10.7)	14	5.0 (3.0–8.2)
*Tb + Tt*	2	0.4 (0.1–1.4)	0	0.0 (0.0–2.7)	2	0.5 (0.2–1.9)	1	0.6 (0.1–3.4)	0	0.0 (0.0–5.7)	1	0.4 (0.1–2.0)
*Tc + Tt*	2	0.4 (0.1–1.4)	0	0.0 (0.0–2.7)	2	0.5 (0.2–1.9)	0	0.0 (0.0–2.3)	0	0.0 (0.0–5.7)	2	0.7 (0.2–2.6)
*Tc + Tv*	1	0.2 (0.04–1.1)	1	0.7 (0.1–4.0)	0	0.0 (0.0–1.0)	0	0.0 (0.0–2.3)	0	0.0 (0.0–5.7)	1	0.4 (0.1–2.0)
*Tt + Tv*	14	2.8 (1.7–4.6)	3	2.2 (0.8–6.2)	11	3.0 (1.7–5.2)	2	1.2 (0.3–4.4)	2	3.1 (0.9–10.7)	10	3.6 (1.9–6.4)

^a^Total infection included cattle that showed both single and coinfections with *Trypanosoma* species.

^b^Subtracting the number of coinfection species gives the number of single *Trypanosoma* infections.

^c^Total infections were calculated by adding the number of single *Trypanosoma* infections after subtraction and the number of coinfections.

*95% CI* 95% confidence interval (lower and upper limit), *n* number of samples.

**Table 5:** Prevalence, number and percentage of *Trypanosoma* spp. in the blood of naturally infected cattle from four different regions in Al Radom National Park, Sudan, detected using PCR.

Prevalence	All animals		Study region							
			Al Radom town		Al Radom Livestock Market		Kafindibei		Murayrayah	
	*n*	% (95% CI)	*n*	% (95% CI)	*n*	% (95% CI)	*n*	% (95% CI)	*n*	% (95% CI)
Tested cattle	509	–								
No. by category			33	–	38	–	268	–	170	–
Total infection^a^	182	35.8 (31.7–40.0)	9	27.3 (15.1–44.2)	19	50.0 (34.9–65.2)	115	42.9 (37.1–48.9)	39	22.9 (17.3–29.8)
*T. brucei* ^b, c^	4	0.8 (0.3–2.0)	0	0.0 (0.0–10.4)	0	0.0 (0.0–9.2)	3	1.1 (0.4–3.2)	1	0.6 (0.1–3.3)
*T. congolense* savannah^b, c^	80	15.7 (12.8–19.1)	0	0.0 (0.0–10.4)	6	1.2 (0.5–2.6)	66	24.6 (19.9–30.1)	8	4.7 (2.4–9.0)
*T. theileri* ^b, c^	68	13.4 (10.7–16.6)	5	15.2 (6.7–30.9)	5	13.2 (5.8–27.3)	36	13.4 (9.9–18.0)	22	12.9 (8.7–18.8)
*T. vivax* ^b, c^	49	9.6 (7.4–12.5)	5	15.2 (6.7–30.9)	9	23.7 (13.0–39.2)	19	7.1 (4.6–10.8)	16	9.4 (5.9–14.7)
Coinfection species	19	3.7 (2.4–5.8)	1	3.0 (0.5–15.3)	1	2.6 (0.5–13.5)	9	3.4 (1.8–6.3)	8	4.7 (2.4–9.0)
*Tb + Tt*	2	0.4 (0.1–1.4)	0	0.0 (0.0–10.4)	0	0.0 (0.0–9.2)	1	0.4 (0.1–2.1)	1	0.6 (0.1–3.3)
*Tc + Tt*	2	0.4 (0.1–1.4)	0	0.0 (0.0–10.4)	0	0.0 (0.0–9.2)	2	0.8 (0.2–2.7)	0	0.0 (0.0–2.2)
*Tc + Tv*	1	0.2 (0.04–1.1)	0	0.0 (0.0–10.4)	0	0.0 (0.0–9.2)	1	0.4 (0.1–2.1)	0	0.0 (0.0–2.2)
*Tt + Tv*	14	2.8 (1.7–4.6)	1	3.0 (0.5–15.3)	1	2.6 (0.5–13.5)	5	1.9 (0.8–4.3)	7	4.1 (2.0–8.3)

^a^Total infection included cattle that showed both single and coinfections with *Trypanosoma* species.

^b^Subtracting the number of coinfection species gives the number of single *Trypanosoma* infections.

^c^Total infections were calculated by adding the number of single *Trypanosoma* infections after subtraction and the number of coinfections.

*95% CI* 95% confidence interval (lower and upper limit), *n* number of samples.

### Insights into trypanocidal drug usage based on questionnaire data

The questionnaire analysis showed that 86.8% (95% CI: 83.6–89.5%) of the screened cattle (*n* = 509) had received trypanocidal drugs within the 30 days before sampling. The treatments included one or more of the following trypanocides: diminazene aceturate, isometamidium chloride and quinapyramine sulphate and chloride. Melarsamine hydrochloride was not reported (Tables 1 and 6). A higher percentage of females (88.4%; 95% CI: 84.8–91.3%) received trypanocidal drugs in the 30 days before sample collection compared to males (82.5%; 95% CI: 75.3–87.9%). Within the three different age categories, the frequency of trypanocidal drug treatments practised by nomads was higher in older cattle than in younger ones, with 89.7% of cattle over three years old being treated in the last 30 days before sample collection. Among the study regions, nomads in Kafindibei treated the highest number of animals, with 268 (100%) receiving treatment during the 30 days before sample collection (Table 1). The questionnaire data also showed that 39.5% (95% CI: 35.3–43.8%) of cattle received a single trypanocidal drug, with diminazene aceturate being the most frequently administered trypanocide, used in 29.7% (95% CI: 25.9–33.8%) of all animals (*n* = 509), as detailed in Table 6. Nomads also practised combination trypanocidal drug treatments, using two or all three of the trypanocides. In total, 47.4% (95% CI: 43.1–51.7%) of all cattle (*n* = 509) received combination treatments in the 30 days before sample collection, with the diminazene aceturate-isometamidium chloride combination being the most frequently used (24.8%; 95% CI: 21.2–28.7%) (Table 6). Factors influencing trypanocidal drug treatments in the 30 days before sample collection, including sex and age of the animals, were analysed using a generalized linear mixed model with herd/*fariq* included as a random effect. No significant correlations were found between these factors at a P-value of < 0.05 (Supplementary Table 3).

**Table 6:** Frequency of trypanocidal drug treatments during a 30-day sample collection period, based on a questionnaire survey, in cattle naturally infected with *Trypanosoma* spp. in Al Radom National Park, Sudan.

Factor	All animals	Trypanocides								
		Animals received a single drug				Animals received drug combinations				
		Total	Berenil	Antrycide	Samorine	Total	B + A	B + S	A + S	B + A + S
No. of the tested cattle	509									
No. (%; 95% CI) of cattle received trypanocides^a^	442 (86.8; 83.6–89.5)	201 (39.5; 35.3–43.8)	151 (29.7; 25.9–33.8)	1 (0.2; 0.04–1.1)	49 (9.6; 7.4–12.5)	241 (47.4; 43.1–51.7)	105 (20.6; 17.3–24.4)	126 (24.8; 21.2–28.7)	1 (0.2; 0.04–1.1)	9 (1.8; 0.9–3.3)
No. (%; 95% CI) of the infected cattle	182 (35.8; 31.7–40.0)	n.a.	n.a.	n.a.	n.a.	n.a.	n.a.	n.a.	n.a.	n.a.
No. (%; 95% CI) of cattle received trypanocides and infected^b^	156 (30.7; 26.8–34.8)	79 (39.3; 32.8–46.2)	60 (39.7; 32.3–47.7)	0 (0.0; 0.0–79.4)	19 (38.8; 26.4–52.8)	77 (32.0; 26.4–38.1)	21 (20.0; 13.5–28.7)	56 (44.4; 36.1–53.2)	0 (0.0; 0.0–79.4)	0 (0.0; 0.0–29.9)
*T. brucei* ^c^	4 (0.8; 0.3–2.0)	2 (1.0; 0.3–3.6)	2 (1.3; 0.4–4.7)	0 (0.0; 0.0–79.4)	0 (0.0–7.3)	2 (0.8; 0.2–3.0)	1 (1.0; 0.2–5.2)	1 (0.8; 0.1–4.4)	0 (0.0; 0.0–79.4)	0 (0.0; 0.0–29.9)
*T. congolense* savannah^c^	74 (14.5; 11.7–17.9)	35 (17.4; 12.8–23.3)	26 (17.2; 12.0–24.0)	0 (0.0; 0.0–79.4)	9 (18.4; 10.0–31.4)	39 (16.2; 12.1–21.4)	4 (3.8; 1.5–9.4)	35 (27.8; 20.7–36.2)	0 (0.0; 0.0–79.4)	0 (0.0; 0.0–29.9)
*T. theileri* ^c^	60 (11.8; 9.3–14.9)	32 (15.9; 11.5–21.6)	25 (16.6; 11.5–23.3)	0 (0.0; 0.0–79.4)	7 (14.3; 7.1–26.7)	28 (11.6; 8.2–16.3)	12 (11.4; 6.7–18.9)	16 (12.7; 8.0–19.6)	0 (0.0; 0.0–79.4)	0 (0.0; 0.0–29.9)
*T. vivax* ^c^	35 (6.9; 5.0–9.4)	21 (10.5; 6.9–15.4)	17 (11.3; 7.2–17.3)	0 (0.0; 0.0–79.4)	4 (8.2; 3.2–19.2)	14 (5.8; 3.5–9.5)	9 (8.6; 4.6–15.5)	5 (4.0; 1.7–9.0)	0 (0.0; 0.0–79.4)	0 (0.0; 0.0–29.9)
Coinfection species	17 (3.3; 2.1–5.3)	11 (5.5; 3.1–9.5)	10 (6.6; 3.6–11.8)	0 (0.0; 0.0–79.4)	1 (2.0; 0.4–10.7)	6 (2.5; 1.2–5.3)	5 (4.8; 2.1–10.7)	1 (0.8; 0.1–4.4)	0 (0.0; 0.0–79.4)	0 (0.0; 0.0–29.9)
*Tb + Tt*	2 (0.4; 0.1–1.4)	1 (0.5; 0.09–2.8)	1 (0.7; 0.1–3.7)	0 (0.0; 0.0–79.4)	0 (0.0–7.3)	1 (0.4; 0.07–2.3)	1 (1.0; 0.2–5.2)	0 (0.0–3.0)	0 (0.0; 0.0–79.4)	0 (0.0; 0.0–29.9)
*Tc + Tt*	2 (0.4; 0.1–1.4)	1 (0.5; 0.09–2.8)	1 (0.7; 0.1–3.7)	0 (0.0; 0.0–79.4)	0 (0.0–7.3)	1 (0.4; 0.07–2.3)	0 (0.0–3.5)	1 (0.8; 0.1–4.4)	0 (0.0; 0.0–79.4)	0 (0.0; 0.0–29.9)
*Tc + Tv*	1 (0.2; 0.04–1.1)	1 (0.5; 0.09–2.8)	0 (0.0–2.5)	0 (0.0; 0.0–79.4)	1 (2.0; 0.4–10.7)	0 (0.0–1.6)	0 (0.0–3.5)	0 (0.0–3.0)	0 (0.0; 0.0–79.4)	0 (0.0; 0.0–29.9)
*Tt + Tv*	12 (2.4; 1.4–4.1)	8 (4.0; 2.0–7.7)	8 (5.3; 2.7–10.1)	0 (0.0; 0.0–79.4)	0 (0.0–7.3)	4 (1.7; 0.7–4.2)	4 (3.8; 1.5–9.4)	0 (0.0–3.0)	0 (0.0; 0.0–79.4)	0 (0.0; 0.0–29.9)

^a^Percentages (%) and the 95% CIs for this raw were calculated from the total number of animals (*n* = 509).

^b^Cattle, both singly infected and coinfected, tested positive for *Trypanosoma* spp. using PCR.

^c^Subtracting the number of coinfection species gives the number of single *Trypanosoma* infections.

*95% CIs* 95% confidence intervals (upper and lower limits), *n.a.* not available.

### Relationship between trypanocidal drug treatments and the occurrence of *Trypanosoma* spp. infections

Questionnaire data revealed that 86.8% of the 509 screened cattle had received either single or combination trypanocidal drugs within the 30 days before sample collection at Al Radom National Park. However, PCR detected that 35.8% (95% CI: 31.7–40.0%) of the tested cattle were infected with *Trypanosoma* spp. (Tables 1 and 6). Comparing the two results showed that 30.7% (95% CI: 26.8–34.8%) of the screened cattle were both infected with *Trypanosoma* spp. and had received trypanocidal drugs, while only 5.1% of *Trypanosoma*-positive cattle had not received trypanocides (Table 6). Unfortunately, *Trypanosoma* spp. were detected not only in animals that had received the curative trypanocide diminazene aceturate alone, but also in most animals treated with isometamidium chloride and quinapyramine sulphate and chloride, either alone or in combination, despite their known curative efficacy and long-lasting prophylactic effect (2–4 months). However, 39.3% (95% CI: 32.8–46.2%) of 201 cattle that received a single trypanocidal drug were positive for *Trypanosoma* spp., and 32.0% (95% CI: 26.4–38.1%) of 241 cattle that received a combination of two or all three trypanocidal drugs were also positive. Diminazene aceturate alone was administered to 151 (29.7% of the total animals), and 39.7% of these were positive for *Trypanosoma* spp., including single infections with *T. brucei*, *T. congolense* savannah and *T. theileri*, as well as coinfection with most of them. The combination treatment of diminazene aceturate and isometamidium chloride was the most frequent (24.8%, 126/509), followed by a combination of diminazene aceturate and quinapyramine sulphate and chloride (20.6%, 105/509). However, 44.4% out of the cattle that received the first combination and 20.0% of the cattle treated with the second combination were positive for *Trypanosoma* spp. (Table 6). Of the nine cattle that had received all three trypanocides, none tested positive for *Trypanosoma* spp. At the species level, *T. congolense* savannah and *T. theileri* were the most frequent *Trypanosoma* species, apparently surviving trypanocidal drug treatments (Table 6). A generalized linear mixed model analysis further supported the apparent trypanocidal drug treatment failure, as no significant correlation was found between treatments and *Trypanosoma* spp. infections (P-value > 0.05) (Table 2).

### Effect of *Trypanosoma* spp. infections and trypanocidal drug treatments on packed cell volume

The mean PCV of all included animals was 28.8% (± 5.9% SD), classifying the animals on average as non-anaemic. Infected cattle showed a slight increase, with a mean PCV of 29.3% (± 5.9% SD) (Table 1 and Supplementary Table 4). Across the three age groups, the PCV values were similar, with the highest mean in infected cattle aged 1 to 3 years, averaging 32.1% (± 7.0% SD). Infected cattle from Al Radom town had the highest mean PCV with 35.4% (± 7.0% SD), compared to the other three study regions (mean PCV range: 28.3–29.8%) (Table 1). The two cattle positives for *T. brucei* only showed the lowest mean PCV of 21.5% (± 2.1% SD), followed by a cow coinfected with *T. congolense* savannah and *T. vivax* with a PCV of 24.0% (± 0.0 SD), and then two cattle coinfected with *T. brucei* and *T. theileri*, with a PCV of 26.5% (± 9.2% SD) (Supplementary Table 4). In total, 85.7% of the animals had a normal PCV, while the rest were anaemic (PCV < 24%). The effect of trypanocidal drug treatments on the odds of having a normal PCV was also analysed using logistic regression. While animals older than three years had significantly lower odds (Odds ratio: 0.430 and 0.417 for bivariable and multivariable models, respectively) of having a normal PCV than animals below one year, a recent treatment with a trypanocide increased the odds to find a normal PCV by 5.59-fold in a multivariable model (Table 7).

**Table 7:** Final logistic regression mixed model identifying variables influencing packed cell volume (PCV) in cattle naturally infected with *Trypanosoma* spp. in Al Radom National Park, Sudan, using herd/*fariq* as a random effect variable.

	Bivariable^a^			Multivariable^b^		
	Odds ratio	95% CI	*P* value	Odds ratio	95% CI	*P* value
Trypanocide use; non-treated vs.						
Treated	5.307	1.041–27.066	0.045	5.593	1.077–29.038	0.041
Age; young (< 1 year) vs.						
1–3 years	0.406	0.159–103.895	0.060	0.416	0.161–1.076	0.070
> 3 years	0.430	0.241–0.765	0.004	0.417	0.233–0.746	0.003
Study region; Murayrayah vs.						
Al Radom town	0.153	0.014–164.652	0.122			
Al Radom Livestock Market	0.476	0.056–406.030	0.497			
Kafindibei	0.709	0.272–185.104	0.483			
Sex; male vs.						
Female	0.568	0.321–1.004	0.052			
*Trypanosoma* spp.; non-infected vs.						
Infected	1.147	0.645–2.042	0.640			

^a^Conditional Nakagawa R^2^ (range): 0.206–0.254; Marginal Nakagawa R^2^ (range): 0.001–0.072.

^b^Conditional Nakagawa R^2^: 0.287; Marginal Nakagawa R^2^: 0.110.

## Discussion

Trypanosomosis remains an economically important disease of cattle in sub-Saharan Africa, including Sudan, with the most significant impact due to infections with *T. congolense*, *T. vivax* and *T. brucei*^2,12,25^. In South Darfur State, trypanosomosis remains a frequently reported disease in cattle in veterinary clinics in tsetse fly zones and beyond, principally in the rainy season (June – October) when animals move north^29,45^. Nevertheless, after Darfur civil war (2003–2007), little is known about the prevalence and distribution of trypanosomosis, particularly in the tsetse fly zone such as Al Radom National Park, and data based on molecular techniques is lacking in particular.

Screening cattle for *Trypanosoma* spp. in Al Radom National Park revealed a prevalence of 35.8%, including single and coinfections. This is higher than previous reports in South Darfur State based on microscopic examination and/or BCT, such as 4.2%^46^ and 7.1%^47^, but lower than recent findings from other parts of Sudan that used more sensitive techniques (e.g. PCR), including Blue Nile State (56.7% and 100%) and West Kordofan (92.3%)^12,25^. However, the 35.8% prevalence is similar to Uganda’s 41.4%, based on ITS-PCR^48^, and higher than the 3.0% in Nigeria using nested ITS-PCR^49^ and 23.8% in Somalia using ITS-PCR^50^. The increase in *Trypanosoma* spp. prevalence over time may be due to improved detection techniques; herein, PCR identified 35.8%, compared to 19.8% with BCT and 3.1% with microscopic examination. Still, the 19.8% BCT prevalence in the present study is higher than the 4.2% reported by Hall, et al.^46^ using the same technique. A second factor contributing to the change in prevalence of *Trypanosoma* spp. in cattle in Al Radom National Park could be the Darfur civil war, which affected vector control programs. As a result, the use of trypanocidal drugs often became the only prophylactic option available, a point raised by nomads during our survey. Moreover, the independence of South Sudan in 2011 had a significant impact, as 40.7% of the interviewed nomads used to migrate to South Sudan during the dry season (March – May). Since the two regions were now in different countries, a concerted and well-managed control strategy for the whole region was no longer available. Additional factors influencing prevalence include ecological differences between the study regions, which significantly affect tsetse fly distribution and habitats^29^, variations in livestock husbandry^51^, the chances for mechanical transmission, particularly due to the abundance of tabanids^7^, the expansion of veterinary services^21^ and the use of trypanocidal/insecticidal drugs as well as the emergence of drug resistance^20,52^. Environmental shifts from rich to poor savannah decrease tsetse habitats, leading to an increase in the mechanical transmission of *Trypanosoma* species over biological transmission. This shift impacts infection types and prevalence, with poorer savannahs having lower prevalence rates^29^. A previous study from a semi-desert zone in Sudan, Khartoum, reported a 4.8% prevalence of *Trypanosoma* spp. using BCT^53^. This is lower than the 19.8% reported in the present study using the same technique, which indicates the impact of different environments.

PCRs identified *T. congolense* savannah, *T. vivax*, *T. brucei* and *T. theileri* in the screened cattle, with *T. congolense* savannah showing the highest prevalence (15.7%). This is consistent with many previous studies in South Darfur State and beyond, where *T. congolense* was most prevalent^12,25,27,46^. Coinfections, including two species, were detected in 3.7% of the animals, with the highest being *T. vivax* and *T. theileri* (2.8%). Coinfections were also detected in cattle in previous studies in Blue Nile and West Kordofan states^12,25^.

Risk factor analysis showed a significant correlation related to the study regions and animal age. Cattle at Al Radom Livestock Market and Kafindibei were significantly more frequently infected than those from Murayrayah. The environment in Kafindibei is rich in watercourses and forests compared to Murayrayah, supporting a favourable habitat for tsetse flies; see the atlas of tsetse flies and bovine trypanosomosis in Sudan^29^. The 50.0% prevalence of *Trypanosoma* spp. in cattle from Al Radom Livestock Market may highlight the impact of the selective nature of nomadic cattle marketing practices. Nomads usually sell adult animals rather than young ones (less than one year old) for better returns or herd replacement. Moreover, they also sell cattle with lower production values, or those that have shown frequent infection or do not respond to treatment^54^. The impact of human activity, particularly the Livestock Market, has been identified as a strong risk factor associated with *Trypanosoma* spp. infection in cattle in Tanzania (*P* = 0.0001)^32^. In the present study, cattle older than three years had significantly (*P* < 0.001) higher *Trypanosoma* spp. infections than those less than one year old. This finding aligns with many previous studies^55–57^. Previous studies indicated that calves showed higher resistance to *Trypanosoma* spp. infections compared to adults, as they exhibited a bone marrow response that resulted in increased erythropoiesis^58,59^. Additionally, calves are kept around the homestead, allowing for better care and closer monitoring for health problems. Finally, with increasing age, the animals have a higher cumulatively chance to be bitten by an infected tsetse fly and, since the infection is not cleared by the immune system, also a higher chance to be positive in the PCR.

While the questionnaire survey primarily focused on the use of trypanocidal drugs, particularly reporting the last trypanocidal drug administered within 30 days before the interview, it also highlighted nomads’ awareness of tsetse flies and trypanosomosis and its symptoms and knowledge of trypanocidal drugs. The high level of awareness underscores the significant economic impact of trypanosomosis as one of the major infectious diseases in cattle in the region. Similar findings were documented in The Gambia, Tanzania and Nigeria, where cattle breeders were aware of tsetse flies, trypanosomosis and drugs^60–62^. Nomads in Al Radom National Park reported using diminazene aceturate, isometamidium chloride and quinapyramine sulphate and chloride, either alone or in combination, to treat and prevent trypanosomosis. In The Gambia, breeders mainly used diminazene aceturate and isometamidium chloride^60^, while those in northern Côte d’Ivoire reported using diminazene aceturate, isometamidium chloride and homidium bromide^63^.

The emergence of resistance to trypanocidal drugs poses significant challenges in controlling animal trypanosomosis. This is primarily due to various related factors, starting from inappropriate diagnosis and continuing with incorrect dosing, the use of inappropriate administration routes and issues related to drug quality from manufacturing to storage. Understanding these factors is crucial for developing effective management and control strategies^20^. In the present study, 86.8% of the cattle had received trypanocidal drugs within 30 days before the interview, highlighting the significant impact of trypanosomosis in Al Radom National Park and the need for improved control strategies. This administration rate was higher than the 50.0% reported in the same region in 1983^46^. Increased drug affordability, awareness of trypanosomosis, changes in infection rates and increasing occurrence of drug resistance may explain this rise. A similar high usage (80.1%) of trypanocidal drugs was found in southwestern Uganda, where cattle received diminazene aceturate, isometamidium chloride or homidium bromide^64^. In Al Radom National Park, 39.5% of cattle received single-drug treatments, with diminazene aceturate being the most common (29.7%), while 47.4% received combination treatments, particularly diminazene aceturate-isometamidium chloride (24.8%). The percentage of single trypanocidal drug treatments was lower than in the Ugandan study by Kasozi, et al.^64^, but diminazene aceturate was similarly popular: 77.2% of surveyed farms in southwestern Uganda treated cattle with a single trypanocidal drug, particularly diminazene aceturate, in the last 30 days before questionnaire survey, and 17.0% of farms used combination trypanocidal drugs, mainly diminazene aceturate-isometamidium chloride. The high use of diminazene aceturate can be attributed to its affordability, availability, anti-inflammatory benefits and a low toxicity risk at the injection site^23^. The high rate of combination use in Al Radom National Park reflects nomads’ lack of confidence in single-drug efficacy, primarily due to the suspected emergence of trypanocidal drug resistance.

Data showed that 30.7% of screened cattle were treated and infected, with *T. congolense* savannah being the most common parasite species. This suggests apparent trypanocidal drug treatment failure, particularly the presence of drug resistance, presumably due to the high frequency of drug usage^20^. However, treatment failures can also have other causes than resistance, in particular underdosing, wrong application or poor quality of the drugs. Substandard veterinary drugs have been reported in sub-Saharan Africa, including Sudan^36,65^. Nomads often obtain veterinary drugs without a prescription or professional supervision, increasing the risk of resistance^36,66^. Diminazene aceturate can protect from infection for up to three weeks^21^. Therefore, *Trypanosoma* spp. positive cattle in the present study that received a diminazene aceturate treatment likely indicate a treatment failure, presumably due to a change in efficacy, although reinfection cannot be ruled out^67^. However, when diminazene aceturate was used in combination with isometamidium chloride and/or quinapyramine sulphate and chloride, which have a known prophylactic effect lasting 2 to 4 months^23,24^, the efficacy of these combinations is obviously changed in South Darfur State. The non-significant association between drug treatment and *Trypanosoma* spp. infections further suggest the treatment failure, and presumably the presence of resistance. A previous study in South Darfur State has reported multi-resistant trypanosomes. However, cattle receiving combination treatments including isometamidium chloride showed no *Trypanosoma* spp.^27^, supporting the use of combination therapy to extend the range of trypanocidal drug efficacy and combat resistance^52^. Similarly, in Kenya, combination therapy was more effective than single trypanocidal treatments^68^.

Anaemia remains one of the key indicators of *Trypanosoma* spp. infection in cattle, typically measured by PCV^69^. The PCV cut-off value for cattle, as defined by Mamoudou, et al.^38^, was used to classify screened cattle as anaemic (PCV < 24%) and non-anaemic (PCV ≥ 24%). In the present study, the mean PCV of infected and non-infected cattle was not significantly different. Remarkably, no correlation was found between infection and PCV. This was consistent with a previous study in the same region by Hall, et al.^46^, who did not find a correlation between the infection and the PCV. The lack of correlation was often attributed to a degree of trypanosome tolerance in *Baggara* cattle. In the present study, recent trypanocidal treatments resulted in a significantly lower chance of animals being anaemic. Presumably, the trypanocides were still effective enough to suppress the trypanosome density to a level that did not cause anaemia, but not enough to lead to a negative PCR result. Coinfection with other pathogens, such as *Babesia* spp. and *Theileria* spp., as well as helminth infections, in particular *Haemonchus* spp., cannot be ruled out as factors influencing the PCV. However, the improper diagnosis and subsequent treatment of the diseased animal relying on trypanocidal drugs only in this situation cannot achieve full curative results, and therefore, the expected health improvement, including an increase in the PCV, does not occur^70^.

## Conclusions

In this study, we used microscopic examination, BCT and PCR to demonstrate that cattle in Al Radom National Park were generally infected with both single and coinfections of *Trypanosoma* species (*T. congolense* savannah, *T. vivax*, *T. brucei* and *T. theileri*), with *T. congolense* savannah being the most prevalent trypanosome. A questionnaire survey highlighted nomads’ awareness of tsetse flies, trypanosomosis and the drugs used for control, which are available in the local markets. The nomads recognised the association between tsetse flies and trypanosomosis, had good knowledge of the clinical signs of trypanosomosis, and practised both single and combination trypanocidal drug treatments using three drugs: diminazene aceturate, isometamidium chloride and quinapyramine sulphate and chloride. National control strategies should be activated for sustainable control of trypanosomosis and its vectors across the endemic regions in Sudan. Moreover, given that nomads migrate with their animals to South Sudan, where tsetse intensity is high, there is an obvious need for a coordinated and well-managed control strategy between both countries. Further studies are needed to confirm the presence of *T. brucei*, as well as studies directly investigating the efficacy of the three trypanocidal drugs and their combinations.

## Supplementary Information

Below is the link to the electronic supplementary material.

Supplementary Material 1

## Data Availability

The relevant information has been included in the manuscript. Data analysed for this manuscript are available from the corresponding author on request.

## Abbreviations

AIC: Akaike information criterion
CI: Confidence interval
BCT: Buffy coat technique
ITS: Ribosomal internal transcribed spacer
PCR: Polymerase chain reaction
PCV: Packed cell volume
