# Supplementary material for: High prevalence of Trypanosoma spp. and apparent trypanocidal drugs inefficacy in cattle in Al Radom National Park, Sudan
Source: Sci Rep. 2026 Jan 27;16:3472. doi: 10.1038/s41598-026-37097-7 (PMC12847771; doi:10.1038/s41598-026-37097-7)

**High prevalence of *Trypanosoma* spp. and apparent trypanocidal drugs inefficacy in cattle in Al Radom National Park, Sudan**

**Khalid M. Mohammedsalih^1,2,3,4^, Maowia M. Mukhtar^5,6^, Abdoelnaim I. Y. Ibrahim^4^, Ibrahim M. I. Mohammedain^4^, Fathel-Rahman Juma^3,4^, Abdelrahim A. Ali^4^, Elwaleed M. Elamin^5^, Ahmed Bashar^4^, Georg von Samson-Himmelstjerna^1,2^, Jürgen Krücken^1,2*^**

^1^Institute for Parasitology and Tropical Veterinary Medicine, Freie Universität Berlin, Robert-von-Ostertag-Str. 7, 14163 Berlin, Germany

^2^Veterinary Centre for Resistance Research, Freie Universität Berlin, 14163 Berlin, Germany

^3^Central Research Laboratory of Darfur Universities, Mousseh district, 63311 Nyala, Sudan

^4^Faculty of Veterinary Science, University of Nyala, Mousseh district, 63311 Nyala, Sudan

^5^Bioscience Research Institute, 11463 Khartoum, Sudan

^6^Institute of Endemic Diseases, University of Khartoum, Qasser Street, P.O. Box 102, Khartoum, Sudan

* Correspondence: [juergen.kruecken@fu-berlin.de](mailto:juergen.kruecken@fu-berlin.de)

**Supplementary Material**

**Supplementary Table 1.** Primers used for PCR amplification of *Trypanosoma* spp. DNA from blood samples of naturally infected cattle in Al Radom National Park, Sudan

**Supplementary Table 2.** Summary of a questionnaire survey on trypanosomosis and its vectors in Al Radom National Park, Sudan.

**Supplementary Table 3.** Risk factors influencing trypanocidal drug treatments during a 30-day sample collection period, based on a questionnaire survey, in cattle naturally infected with *Trypanosoma* spp. in Al Radom National Park, Sudan.

**Supplementary Table 4.** Comparison of mean packed cell volume (PCV) among cattle naturally infected with *Trypanosoma* spp. in Al Radom National Park, Sudan.

**Supplementary Fig. 1.** Study regions in Al Radom National Park, South Darfur State, Sudan.

**Supplementary Tables**

**Supplementary Table 1.** Primers used for PCR amplification of *Trypanosoma* spp. DNA from blood samples of naturally infected cattle in Al Radom National Park, Sudan

| **Type** | **Name** | | **Primer sequence (5′— 3′)** | ***Trypanosoma* (*T*.)** | **Size (bp)** | **Annealing temperature (°C)** | **Reference** |
| --- | --- | --- | --- | --- | --- | --- | --- |
| Pan-Trypanosoma specific | ITS | 1 | GATTACGTCCCTGCCATTTG | *T. congolense* | 1408 – 1501 | 55 | 11 |
|  |  | 2 | TTGTTCGCTATCGGTCTTCC | *T. brucei* | 1215 |  |  |
|  |  | 3 | GGAAGCAAAAGTCGTAACAAGG | *T. theileri* | 998 |  |  |
|  |  | 4 | TGTTTTCTTTTCCTCCGCTG | *T. simiae* Tsavo | 951 |  |  |
|  |  | |  | *T. simiae* | 847 |  |  |
|  |  | |  | *T. vivax* | 620 |  |  |
| Mono-specific | Tvw | | CTGAGTGCTCCATGTGCCAC | *T. vivax* | 150 | 60 | 5 |
|  |  | | CCACCAGAACACCAACCTGA |  |  |  |  |
|  | TCS | | CGAGCGAGAACGGGCAC | *T. congolense* savannah | 321 | 60 | 14 |
|  |  | | GGGACAAACAAATCCCGC |  |  |  |  |
|  | TCF | | GGACACGCCAGAAGGTACTT | *T. congolense* forest type | 350 | 60 | 15 |
|  |  | | GTTCTCGCACCAAATCCAAC |  |  |  |  |
|  | TBR | | CGAATGAATATTAAACAATGCGCAG | *T. brucei* sensu-lato (subspecies) | 177 | 58 | 16 |
|  |  | | AGAACCATTTATTAGCTTTGTTGC |  |  |  |  |
|  | Tgs | | GCTGCTGTGTTCGGAGAGC | *T. brucei gambiense* | 308 | 63 | 17 |
|  |  | | GCCATCGTGCTTGCCGCTC |  |  |  |  |
|  | TMS | | CCGGTCAAAAACGCATT | *T. simiae* | 437 | 60 | 5 |
|  |  | | AGTCGCCCGGAGTCGAT |  |  |  |  |
|  | CATL | | CGTCTCTGGCTCCGGTCAAAC | *T. theileri* | 273 | 65 | 18 |
|  |  | | TTAAAGCTTCCACGAGTTCTTGATGATCCAGTA |  |  |  |  |
|  | MP | | CAACGACAAAGAGTCAGT | *T. evansi* | 373 | 55 | 19 |
|  |  | | ACGTGTTTTGTGTATGGT |  |  |  |  |

**Supplementary Table 2.** Summary of a questionnaire survey on trypanosomosis and its vectors in Al Radom National Park, Sudan.

| **Variables** | **Total respondents** | **Frequency** | **Proportion (%)** |
| --- | --- | --- | --- |
| Animal husbandry |  |  |  |
| Nomadic system | 27 | 24 | 88.9 |
| Sedentary farming | 27 | 3 | 11.1 |
| Herd composition |  |  |  |
| Single species (cattle only) | 27 | 2 | 7.4 |
| Mixed species (e.g. cattle, sheep and goats) | 27 | 25 | 92.6 |
| Contact with wildlife (during the last year) |  |  |  |
| Yes | 27 | 24 | 88.9 |
| No | 27 | 3 | 11.1 |
| Cross-border grazing (South Sudan) in the last two years |  |  |  |
| Yes | 27 | 11 | 40.7 |
| No | 27 | 16 | 59.3 |
| Know tsetse flies (*Glossina* spp.) |  |  |  |
| Yes | 27 | 27 | 100 |
| No | 27 | 0 | 0 |
| Last sighting of tsetse flies |  |  |  |
| <6 months | 27 | 14 | 51.9 |
| 6 – 12 months | 27 | 5 | 18.5 |
| >1 year | 27 | 8 | 29.6 |
| Know trypanosomosis |  |  |  |
| Yes | 27 | 27 | 100 |
| No | 27 | 0 | 0 |
| Know trypanosome transmission |  |  |  |
| Tsetse flies | 27 | 27 | 100 |
| Tabanids | 27 | 0 | 0 |
| Others (e.g. ticks) | 27 | 0 | 0 |
| Diagnosis of trypanosomosis |  |  |  |
| Self-diagnosis | 27 | 27 | 100 |
| Paravets | 27 | 0 | 0 |
| Veterinarians | 27 | 0 | 0 |
| Clinical symptoms of trypanosomosis in cattle |  |  |  |
| Addressed no symptoms^a^ | 27 | 0 | 0 |
| Addressed only one symptom^a^ | 27 | 0 | 0 |
| Addressed two symptoms^a^ | 27 | 0 | 0 |
| Addressed more than two symptoms^a^ | 27 | 27 | 100 |
| Treatment and control of trypanosomosis |  |  |  |
| Trypanocidal drugs alone | 27 | 0 | 0 |
| Trypanocidal drugs and tsetse fly control (including one or more of the following practices: moving to a new area, cleaning shelters, burning dung, using smoke and/or applying pour-on insecticides) | 27 | 27 | 100 |

^a^ Clinical symptom of cattle trypanosomosis: fever, anorexia, pale mucus membrane, poor body condition, rough coat, reduced milk production, emaciation and death.

**Supplementary Table 3.** Risk factors influencing trypanocidal drug treatments during a 30-day sample collection period, based on a questionnaire survey, in cattle naturally infected with *Trypanosoma* spp. in Al Radom National Park, Sudan.

|  | Bivariable^a^ | | | Multivariable^b^ | | |
| --- | --- | --- | --- | --- | --- | --- |
|  | Odds ratio | 95% confidence interval | P value | Odds ratio | 95% confidence interval | P value |
| Sex; male *vs.* |  |  |  |  |  |  |
| Female | 1.687 | 0.360 – 7.916 | 0.663 | 1.720 | 0.302 – 9.804 | 0.542 |
| Age; young (<1 year) *vs.* |  |  |  |  |  |  |
| 1–3 years | 2.261 | 0.346 – 14.750 | 0.394 | 2.554 | 0.331 – 19.698 | 0.369 |
| >3 years | 2.623 | 0.596 – 11.536 | 0.202 | 2.570 | 0.521 – 12.673 | 0.246 |
| PCV; non-anaemic (≥24%) *vs.* |  |  |  |  |  |  |
| Anaemic (<24%) | 112.087 | 0.849 – 14799.790 | 0.058 | 121.012 | 0.889 – 16471.180 | 0.056 |

^a^ Conditional Nakagawa R^2^ (range): 0.977 – 0.981; Marginal Nakagawa R^2^ (range): 0.000 – 0.016.

^b^ Conditional Nakagawa R^2^: 0.982; Marginal Nakagawa R^2^: 0.016.

**Supplementary Table 4.** Comparison of mean packed cell volume (PCV) among cattle naturally infected with *Trypanosoma* spp. in Al Radom National Park, Sudan.

| **Factor** | **All animals** | **PCV (%)** |
| --- | --- | --- |
| No. of the tested cattle | 509 | 28.8±5.9 |
| Non-infected | 327 | 28.5±5.8 |
| *Trypanosoma* infected^a^ | 182 | 29.3±5.9 |
| *T. brucei*^b^ | 2 | 21.5±2.1 |
| *T. congolense* savannah^b^ | 77 | 28.4±5.0 |
| *T. theileri*^b^ | 50 | 31.1±6.6 |
| *T. vivax*^b^ | 34 | 28.7±6.1 |
| *Tb+Tt* | 2 | 26.5±9.2 |
| *Tc+Tt* | 2 | 32.5±3.5 |
| *Tc+Tv* | 1 | 24.0±0.0 |
| *Tt+Tv* | 14 | 31.2±6.0 |

^a^ Includes animals infected with a single or two *Trypanosoma* species.

^b^ Coinfected animals were not included. PCV values are means of two replicates of the same sample and are expressed as mean ± standard deviation (SD).

**Supplementary Figures**

**Supplementary Fig. 1.** Study regions in Al Radom National Park, South Darfur State, Sudan.


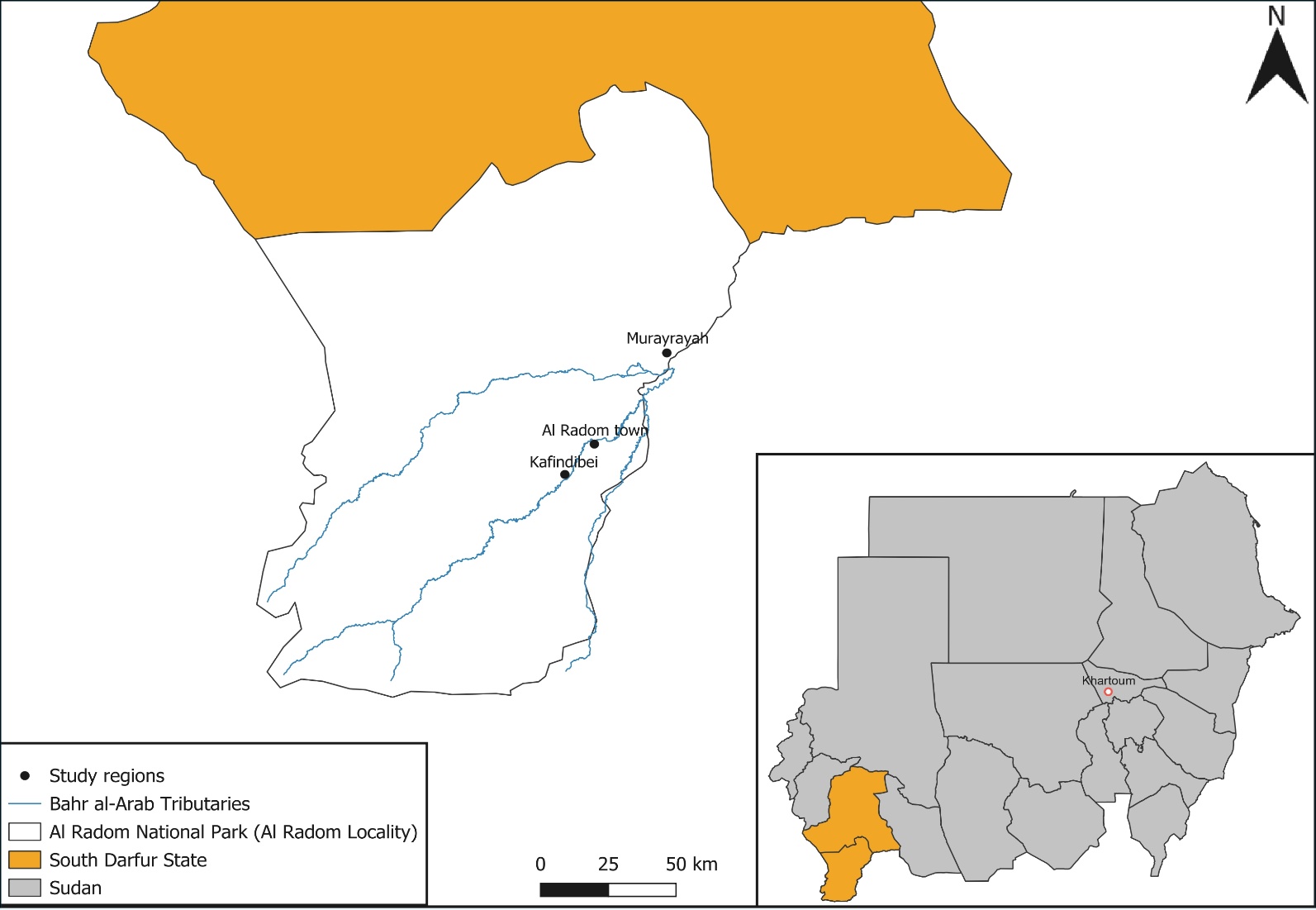

Supplement: Supplementary file 1 — Supplementary Material 1 [file 41598_2026_37097_MOESM1_ESM.docx]
